# Spontaneous supratentorial intracerebral hemorrhage: Does surgery benefit comatose patients?

**DOI:** 10.4103/0972-2327.70881

**Published:** 2010

**Authors:** Cem Yilmaz, Serdar Kabatas, Salih Gulsen, Tufan Cansever, Doga Gurkanlar, Hakan Caner, Nur Altinors

**Affiliations:** Department of Neurosurgery, Baskent University, Ankara, Turkey

**Keywords:** Mortality, outcome, spontaneous supratentorial intracerebral hemorrhage, surgery, treatment

## Abstract

**Introduction:**

Treatment of spontaneous supratentorial intracerebral hemorrhage (SICH) is still controversial. We therefore analyzed the comatose patients diagnosed as having spontaneous SICH and treated by surgery.

**Materials and Methods:**

We retrospectively analyzed the collected data of 25 comatose patients with initial Glasgow Coma Scale (GCS) ≤ 8 diagnosed as having spontaneous SICH and they had been treated by surgical evacuation between 1996 and 2008. The outcome was assessed using Glasgow outcome scale (GOS). The side and location of the hematoma and ventricular extension of the hematoma were recorded. The hematoma volume was graded as mild (<30 cc), moderate (30–60 cc) and massive (>60 cc).

**Results:**

Age of the patients ranged from 25 to 78 years (mean: 59.6 ± 15.14 years). Among the 25 patients studied, 11 (44%) were females and 14 (56%) were males. GCS before surgery was <5 in 8 (32%) patients and between 5 and 8 in 17 (68%) patients. The hematoma volume was less than 30 cc in 2 patients, between 30 and 60 cc in 9 patients and more than 60 cc in 14 patients. Fourteen of the patients had no ventricular connection and 11 of the hematomas were connected to ventricle. All the 25 patients were treated with craniotomy and evacuation of the hematoma was done within an average of 2 hours on admission to the emergency department. Postoperatively, no rebleeding occurred in our patients. The most important complication was infection in 14 of the patients. The mortality of our surgical series was 56%. GCS before surgery was one of the strongest factors affecting outcome GCS (oGCS) (*P* = 0.017). Income GCS (iGCS), however, did not affect GOS (*P* = 0.64). The volume of the hematoma also affected the outcome (*P* = 0.037). Ventricular extension of the hematoma did affect the oGCS and GOS (*P* = 0.002), but not the iGCS of the patients (*P* = 0.139).

**Conclusion:**

Our data suggest that being surgically oriented is very important to achieve successful outcomes in a select group of patients with SICH.

## Introduction

Intracerebral hemorrhage (ICH) refers to spontaneous nontraumatic bleeding from intraparenchymal blood vessels. It is a major public health problem causing high morbidity and mortality. It accounts for 10–15% of all strokes in the United States and Europe and 20–30% in Asian populations.[1–7] Additionally, nearly half of the patients with ICH die and many of the survivors may be severely disabled.[2,8] Although there have been very important advances in early diagnosis of ICH by computerized tomography (CT), improvements in neuroanesthesia and neurosurgical critical care, and development of microscope-guided surgical techniques, the efficacy of surgical treatment for primary ICH is still controversial.[6,9,10] Also, the rate and indication for surgery shows significant differences internationally reflecting this controversy.[3] Comatose patients [Glasgow coma scale (GCS) ≤ 8] with ICH constitute a very special subgroup because of the high mortality rate and uncertainty of the best therapeutic approach. We therefore aimed to analyze the records of 25 comatose patients with initial GCS ≤ 8 diagnosed as having spontaneous supratentorial intracerebral hemorrhage (SICH) and treated by surgical evacuation.

## Materials and Methods

The hospital records of 25 consecutive patients with GCS ≤ 8 who underwent surgical evacuation for spontaneous SICH at the Department of Neurosurgery of Baskent University, between 1996 and 2008, were reviewed in this study. Those with traumatic hemorrhages, hemorrhages due to tumors, and vascular malformations were excluded from the study. Patients with cerebellar and brain stem hemorrhages and pediatric patients were also excluded from the study.

All the patients were first evaluated by emergency room physicians, immediately followed by confirmation of diagnosis of ICH using a baseline non-contrast cranial CT seen by one of the four staff neurosurgeons. Complete physical and neurological examination was made and medical history of the patient including both the present situation and previous illnesses was carefully noted. Drug usage with special attention to anticoagulant and antiplatelet medicines was also noted. CT angiography, magnetic resonance imaging (MRI) and digital subtraction angiography (DSA) were done to rule out tumor hemorrhage or underlying vascular malformation. The decision for surgery or conservative treatment was made by the neurosurgeon after sharing information and opinions with the patient’s family or first-degree relatives if possible.

Consciousness level at admission was evaluated using the GCS and patients with a score >8 were excluded from the study. Outcome was assessed using the Glasgow outcome scale (GOS; GOS 1: death, GOS 2: vegetative state, GOS 3: severe disability, GOS 4: moderate disability, GOS 5: good recovery). The side and location of the hematoma and ventricular extension of the hematoma were recorded. Volume of the hematoma was calculated by multiplying the greatest transverse, sagitt al and coronal diameters and dividing the product by two. Hematoma volume was graded as mild (<30 cc) moderate (30–60 cc) and massive (>60 cc).

### Statistical analysis

Data were analyzed by SPSS version 13.0 software. The effects of the variables (age, gender, income GCS (iGCS), volume of the hematoma, ventricular extension and comorbidities) on the iGCS and the results [outcome GCS (oGCS) and GOS] were analyzed using a linear regression test. Significance was set at *P* < 0.05 with 95% confidence intervals.

## Results

Among the 25 patients studied, 11 (44%) were females and 14 (56%) were males, aged between 25 and 78 years (mean: 59.6 ± 15.14). Age groups were as follows: 8 patients were younger than 55 years (32%), 7 patients were between 56 and 65 years (28%) and 10 patients were between 66 and 78 years (40%). Regarding previous medical history, 18 of them were hypertensive (72%), and 6 of them had diabetes mellitus type II (24%). Seven of the patients had coronary artery disease, 7 had cerebrovascular accident history and 6 had chronic renal failure. Only 2 patients had an unremarkable medical history.

The iGCS was <5 in 8 (32%) and between 5 and 8 in 17 (68%) of the patients. The hematoma volume was less than 30 cc in 2 patients, between 30 and 60 cc in 9 patients and more than 60 cc in 14 patients. Fourteen of the patients had no ventricular connection and 11 of the hematomas were connected to ventricle. All the 25 patients were treated with craniotomy and evacuation of the hematoma was done within an average of 2 hours on admission to the emergency department. Comorbidities of the patients seemed not to affect the iGCS, oGCS and GOS (*P* >0.05) [Table 1].

**Table 1:** Comorbidities of the patients and their effects on iGCS, oGCS and GOS

	Diabetes mellitus	Hypertension	Cerebrovascular attack	Coronary disease	Chronic renal failure
iGCS	0.674	0.284	0.539	0.291	0.112
oGCS	0.870	0.398	0.686	0.126	0.919
GOS	0.656	0.426	0.927	0.226	0.775
Number of patients (%)	24	78	28	28	24

Significance was set at *P* < 0.05. Comorbidities of the patients seemed not to affect the iGCS, oGCS and GOS (*P* > 0.05), respectively.

Postoperatively, no rebleeding occurred in our patients. The most important complication was infection in 14 of the patients. Pneumonia (six patients), urinary tract infection (two patients), meningitis (one patient), sepsis (two patients) and mixed infection (three patients) were the infections that occurred in our patients. The mortality in our surgical series was 56%. Sixteen patients died and one patient was discharged in a vegetative state (4%), and four patients were severely disabled (20%). Moderate disability and good recovery were achieved in four of the patients (20%). Factors that could have affected surgery outcome based on mortality and GOS at the time of discharge from the hospital were studied [Table 2].

**Table 2:** Characteristics of the patients, volume and the ventricular extension of hematoma, and their effects on iGCS, oGCS and GOS

	Age	Gender (Female/Male)	iGCS	Volume	Ventricular extension (±)
iGCS	0.919	0.759	–	0.153	0.139
oGCS	0.554	0.702	0.017#	0.069	0.002#
GOS	0.406	0.523	0.064	0.037#	0.002#
Mean ± SD, ratio and percentage	59.56 ± 15.14	11/14	5.8 ± 1.75	72 ± 38.26	44%

SD, standard deviation. Significance was set at *P* < 0.05;

# iGCS did affect the oGCS (*P* = 0.017). The volume of the hematoma did affect GOS (*P* = 0.037). The ventricular extension of the hematoma did affect the oGCS and GOS (*P* = 0.002).

Gender seems not to affect the clinical presentation and the outcome (iGCS = 0.759; oGCS = 0.702; GOS = 0.523). Age also did not affect the mortality in our groups (iGCS = 0.919; oGCS = 0.554; GOS = 0.406). Hypertension was the most commonly seen disease in patients with ICH (78%), but it did not have any effect on outcome (iGCS = 0.398; oGCS = 0.426; GOS = 0.284). GCS before surgery was one of the strongest factors affecting oGCS (*P* = 0.017). The iGCS, however, did not affect GOS (*P* = 0.64). Volume of the hematoma also affected the outcome. The volume of the hematoma did affect GOS (*P* = 0.037), but not the iGCS (*P* = 0.153) and oGCS (*P* = 0.069). Ventricular extension of the hematoma did affect the oGCS and GOS (*P* = 0.002), but not the iGCS of the patients (*P* = 0.139).

## Discussion

Although the signs and symptoms of intracerebral hematoma seem to be mostly related to compressive and destructive effects of the rapidly developing mass, the pathophysiology of ICH definitely extends beyond that. Developing cerebraledema, perilesional ischemia and the toxic effects of components of the hematoma are reported as causes of secondary injury.[11–13]

Perihematomaledema developing rapidly after ICH increases the intracranial pressure and can result in herniation. This early phase involves hydrostatic pressure and clot retraction with movement of serum from the clot into the surrounding tissue, the second phase is related to coagulation cascade and thrombin production and the third phase is related to erythrocyte lysis and hemoglobin toxicity.[14,15] Hemoglobin and its breakdown products, heme and iron, are shown to be neurotoxic through mechanisms involving iron-catalyzed production of reactive oxygen species.[11]

Cerebral blood flow is reduced in the perihematomal region in both experimental and clinical studies. It is not clear if this is an ischemic penumbra, functionally impaired but potentially salvageable tissue or a primary reduction in cerebral metabolism due to mitochondrial dysfunction because of the toxic affects of blood degradation products.[5,16–19] Whether or not this is an ischemic penumbra or primary reduction in cerebral metabolism, theoretically evacuation of clot mass as much as possible with maximal preservation of neurological function should definitely improve the outcome and this is the most important rationale supporting surgery.[20] The first report published in 1961 by McKissock and colleagues and many following clinical studies showed no overall benefit of surgery for the treatment of ICHs. In the study by McKissock, surgically treated patients with GCS < 8 and hematoma volume > 60 cc had a mortality rate of 91%. The largest of the studies was *The Surgical Trial in Intracerebral Hemorrhage* (STICH) published in 2005 by Mendelow and colleagues.[18–21] This study excluded patients having GCS < 5 and patients having GCS between 5 and 8 had a rate of unfavorable outcome 91%. This study also showed that patients with spontaneous SICH in neurosurgical units showed no overall benefit from early surgery when compared with initial conservative treatment.

The mortality of our surgical series was 56% and the rate of an unfavorable outcome 80%. The mortality rate was lower than that reported in most of the clinical series.[21] We analyzed the results of this study and found out that it is mostly due to the selection of patients for surgery as certain subgroups in our series had a higher mortality rate. Twelve of our 25 patients had a GCS ≤ 5 and this subgroup had a mortality of 83.3%. Hematoma volume is a powerful predictor of 30-day mortality in patients with ICH (*P* = 0.037). Hematoma volume adds to intracranial volume and may lead to life-threatening elevation of intracranial pressure.[22–24] The hematoma volume was also more than 60 cc in 18 patients and mortality of this subgroup was 77.8%, but the mortality rate was 28.6% in the patients who had hematoma volume less than 60 cc. Similarly, Broderick and colleagues in 1993 reported that patients with a parenchymal hemorrhage volume of 60 cc or more on their initial CT and a GCS score of 8 or less had a predicted 30-day mortality of 91%.[23]

Age was reported to be one of the factors predicting the outcome of surgery. In our series, the highest mortality rate was observed for patients younger than 55 years (mortality 83%), followed by patients between 66 and 85 years of age (mortality 70%). Patients between 56 and 65 years had the best outcome (mortality 50%). Elderly patients would be expected to have poor outcome from surgery, but it was surprising that the youngest age group had the worst outcome. The reason for this was that these patients had poorer GCS scores on admission (five of eight patients having a GCS ≤ 5 on admission). Even if the GCS of young patients is low, they are more likely to have surgery as neurosurgeons usually feel that they would have a better outcome as they have less comorbidity, which is an important factor in deciding upon surgery for treatment of ICH. The families of younger patients are also more likely to request for surgery.

Gender did not affect the outcome in our series. Although estrogen is a well-known neuroprotective hormone, most of the clinical studies showed that gender did not affect outcome of ICH and other type of strokes, which is a more sensible result when the average age of the stroke patients is taken into consideration.[25–27] Ventricular extension of the hematoma was reported to be an indicator of poor prognosis.[2,28,29] In our series, we found that it did have a significant effect on the outcome (*P* = 0.002).

In this study, we analyzed the surgical outcome of 25 comatose (GCS ≤ 8) patients with spontaneous SICH treated with surgery. Our study has some limitations due to the less number of patients and it also does not compare the outcome with that of conservative management. These results, however, may still be valuable as they are the results of a single center’s consecutive patients and it suggests that being surgically oriented may result in a better outcome in selected patients.
